# Mutational and splicing landscape in a cohort of 43,000 patients tested for hereditary cancer

**DOI:** 10.1038/s41525-022-00323-y

**Published:** 2022-08-25

**Authors:** Carolyn Horton, Ashley Cass, Blair R. Conner, Lily Hoang, Heather Zimmermann, Nelly Abualkheir, David Burks, Dajun Qian, Bhuvan Molparia, Huy Vuong, Holly LaDuca, Jessica Grzybowski, Kate Durda, Robert Pilarski, Jessica Profato, Katherine Clayback, Martin Mahoney, Courtney Schroeder, Wilfredo Torres-Martinez, Aaron Elliott, Elizabeth C. Chao, Rachid Karam

**Affiliations:** 1 Ambry Genetics. One Enterprise, Aliso Viejo, CA 92656 USA; 2 Roswell Park Comprehensive Cancer Center, 665 Elm St, Buffalo, NY 14203 USA; 3 Indiana University School of Medicine, 975 W. Walnut Street, IB 130, Indianapolis, IN 46202 USA; 4 Realm IDx. One Enterprise, Aliso Viejo, CA 92656 USA; 5 University of California, Irvine, School of Medicine, 1001 Health Sciences Rd, Irvine, CA 92617 USA

**Keywords:** Genetic testing, Cancer genetics, RNA splicing

## Abstract

DNA germline genetic testing can identify individuals with cancer susceptibility. However, DNA sequencing alone is limited in its detection and classification of mRNA splicing variants, particularly those located far from coding sequences. Here we address the limitations of splicing variant identification and interpretation by pairing DNA and RNA sequencing and describe the mutational and splicing landscape in a clinical cohort of 43,524 individuals undergoing genetic testing for hereditary cancer predisposition.

Nearly all clinically available multigene panel tests (MGPT) focus on the analysis of protein-coding exons of DNA with little coverage in the introns^1,2^. Clinically significant variants within the intron predominantly impact splicing; therefore, a splicing profile by RNA sequencing (RNA-seq) can improve the detection of pathogenic variants while also providing functional evidence for accurate interpretation of putative splicing variants^3–6^. However, published evidence on the utility of RNA-seq has been limited by studies with small sample size, highly selected cohorts, and those conducting RNA-seq in follow-up to uninformative DNA results^6–10^.

In this study, 43,599 tests from 43,524 consecutive individuals who underwent paired DNA-RNA genetic testing from March 2019 through April 2020 were eligible for analysis. A total of 15,288 reported variants, including Pathogenic (P) (*n* = 4565), Likely Pathogenic (LP) (*n* = 565), and Variant of Unknown Significance (VUS) (*n* = 10,158), were reported in 18 RNA-covered genes (Supplemental Table 1) amongst 12,859 cases. We found that while missense variants were most observed (69.2% of variants), splicing variants were not rare (6.2%), occurring more than twice as often as copy number variations such as gross deletions/duplications (2.8%) (Fig. 1a). The classification of unique splicing variants (*n* = 555 out of 7136 unique total variants) was determined based on their position with respect to the exon (Fig. 1b). A minority of exonic variants were classified as P/LP (22.7%) and most were classified as VUS (77.3%); however, exonic variants located at the last nucleotide of the exon were more often P/LP (62.7%) due to their impact on splicing. With regards to intronic variants, the vast majority of variants at the well conserved canonical acceptor and donor splice-site (positions −1, −2, and +1, +2 respectively) were classified as P/LP (95.6%). Importantly, 68 P/LP variants located beyond the consensus splice-site (more than 5 nucleotides from the exon) were identified.

**Fig. 1:**
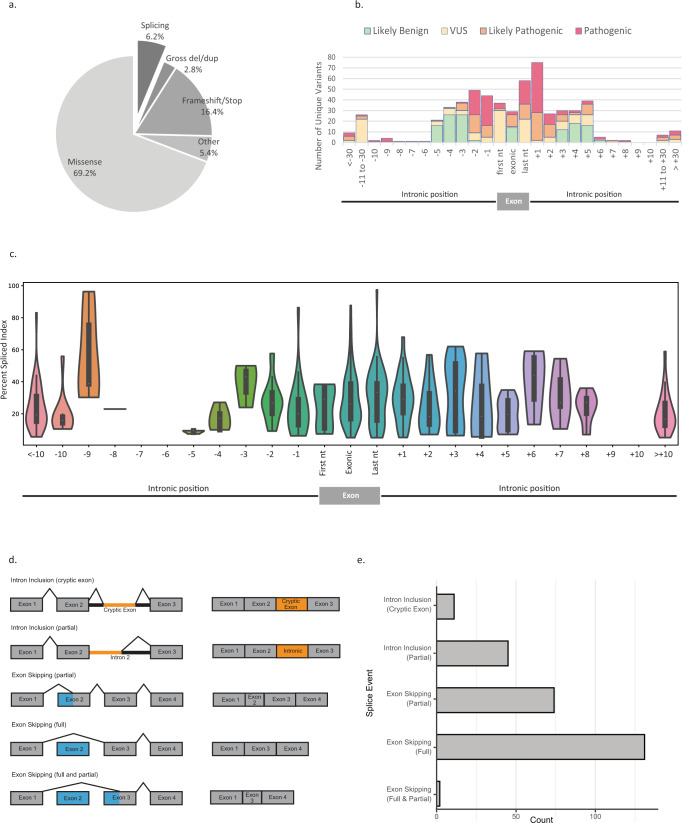
Splicing landscape of ~43,000 patients receiving paired DNA-RNA genetic testing. **a** Distribution of variant type for variants with classification of VUS, LP, or P (*N* = 15,288 reported DNA variants); **b** Splicing variant classification by nucleotide position (*N* = 555 unique splicing variants among 7136 unique total DNA variants); **c** Violin plots demonstrating the percent spliced index of splicing events associated with variants (P/LP/VUS) in patients (*N* = 516 DNA/RNA associated variants), with comparison to their median in healthy donor controls shown in Supplemental Fig. 1. Within each violin, the white dot represents the median, the boundaries of the thin black rectangle represent the interquartile range, and the black line extending from the rectangle represents 1.5× interquartile range; **d** Schematic of the splicing event types considered (gold, inclusion of intronic sequence; blue, exclusion of exonic sequence); **e** Distribution of splicing events associated with reportable variants by splicing event type (*N* = 263 unique transcripts). Variant of Unknown significance (VUS), Likely Pathogenic (LP), Pathogenic (P), nucleotide (nt), Percent Spliced Index (PSI), Deletion/Duplication (del/dup).

There were 263 unique splicing events associated with a DNA variant identified across the 18 genes studied. As some transcripts can be associated with several DNA variants, these corresponded to 516 total combinations of unique DNA variants with associated splicing events across individuals. The association between a splice event and a given variant was established after consideration of the percent splice index (PSI, or the relative percentage of alternative/abnormal transcript)^11^, the specificity of the splice event (i.e., absence of the event at similar PSI in healthy controls), the reproducibility of the splice impact in additional carriers and/or by other functional assays, and the potential mechanism underlying the variant’s impact on splicing (e.g., creation of a novel splice site, weakening of a native splice site, deletion of a branch point, or disruption of a potential exonic splicing enhancer). Analysis of the variant distribution indicates that deleterious splicing DNA variants result in elevated PSI levels of abnormal transcripts compared to controls regardless of the nucleotide position (Fig. 1c, Supplementary Fig. 1). Most splicing events associated with DNA variants resulted in partial or full exon skipping, observed in 205 individuals (Fig. 1d, e). However, intron inclusion events were also associated with 45 variants leading to partial intron inclusion and 11 variants resulting in the inclusion of intronic sequences into cryptic exons. Notably, variants leading to inclusion of cryptic exons are located outside of our standard clinical reporting range for DNA MGPT and would not have been detected without RNA-seq. We found that RNA-seq allowed for the characterization of alternative splicing events and improved our understanding of the various mechanisms of pathogenicity associated with aberrant splicing.

Evidence obtained from RNA-seq impacted variant classification in 549 individuals with potential splicing variants. The RNA impact on classification of potential splicing variants based on nucleotide position is shown in Fig. 2a, b (intronic variants classified as B/LB prior to this study that had no aberrant RNA were excluded). Splicing variants were defined as those with a deleterious impact by in silico modeling, any intronic variant within five nucleotides of the exon, and variants with abnormal RNA transcript detected. RNA impacted variants include those in which RNA evidence led to a reclassification but exclude those in which RNA evidence was concordant with an existing interpretation but did not prompt reclassification. RNA was especially informative for upgrades of DNA variants at intronic positions +3 or +5 (*n* = 33), intronic variants beyond 10 nucleotides from the exon (*n* = 24), and variants at the last nucleotide of the exon (*n* = 36). Intronic variants not impacted by RNA evidence (Fig. 2b) were more likely to already be classified as P/LP before RNA-seq was performed. Alterations classified as VUS >10 nucleotides from the exon had an associated RNA variant, but RNA and/or other clinical evidence was not sufficient for reclassification to P/LP. In addition to splicing variants, interpretation of gross duplications is also aided by RNA-seq by determining if a duplication occurs in tandem with the original sequence, as detailed previously^12^ and reinforced within this dataset via four gross duplications that were reclassified from VUS to P/LP. Incorporation of RNA-seq led to an absolute increase in the positive yield of 0.2% (*n* = 87 individuals) and absolute decrease in VUS rate of 0.7% (*n* = 305 individuals).

**Fig. 2:**
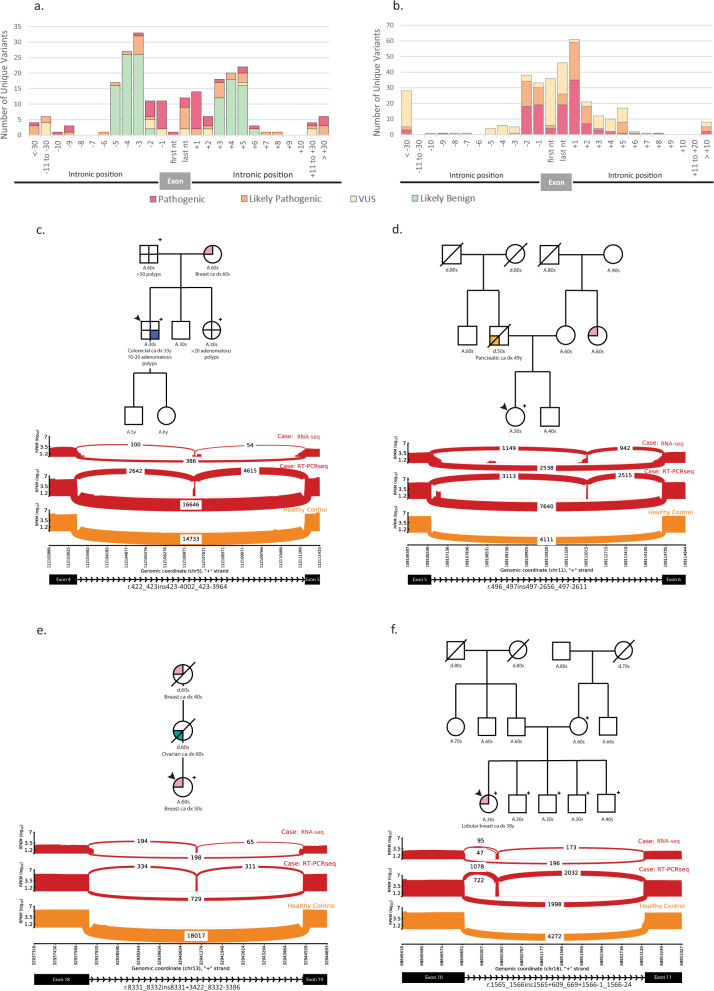
Identification of clinically significant splicing variants. **a** Classification by nucleotide position of splicing variants whose classification was impacted by RNA (classification as assessed without RNA evidence differs from classification with RNA evidence, *N* = 549 variants); **b** Classification by nucleotide position of splicing variants with no RNA impact (classification did not depend on RNA evidence, *N* = 523 variants); **c**–**f** Representative deep-intronic variants. Pedigrees in panels **c**–**f** describe personal and family cancer/polyp history. Sashimi plots in panels **c**–**f** include RT-PCRseq and capture RNA-seq data and represent relevant alignments and exon junction-spanning reads as arcs with read counts displayed. Alternative splicing events were absent in healthy controls. **c** Case description of an individual with likely pathogenic (LP) variant *APC* c.423-3958C > T. Familial testing identified the variant in the proband’s sister and father. Sashimi plots depict the inclusion of a cryptic exon caused by this variant. **d** Case description of an individual with pathogenic variant *ATM* c.497-2661A > G. Familial testing has not been performed. Sashimi plots depict the inclusion of a cryptic exon caused by this variant. **e** Case description of an individuals with LP variant *BRCA2* c.8332-3384A > T. Familial testing has not been performed. Sashimi plots depict the inclusion of a cryptic exon caused by this variant. **f** Case description of an individual with LP variant *CDH1* c.1565 + 672_1566-23del2827. Familial testing identified the variant in all siblings. Sashimi plots depict intron retention events caused by the deletion.

We utilized RNA-seq to identify novel deep intronic P/LP variants without the need for DNA sequencing of the entire intron. DNA variants were only reported in this region when there was an abnormal RNA detected. This allows for increased detection of clinically relevant variants without the substantial VUS burden that results from DNA-only sequencing of intronic regions. We found that 12.3% (*n* = 45) of individuals with intronic P/LP variants had variants located >10 nucleotides from the exon (frequency of one *per* 968 patients tested), and 7.7% (*n* = 28) had variants located >20 nucleotides from the exon (one *per* 1554 patients tested). The clinical history and RNA-seq data for selected individuals with deep intronic variants are shown in Fig. 2c–f. These variants were all predicted by SpliceAI^13^ to strengthen relatively weak cryptic donor or acceptor splice sites within the intron. The *APC* c.423-3958C > T LP variant (Fig. 2c) converts C to T in the +6 position of a cryptic splice donor site, which strengthened the cryptic site and led to the inclusion of a cryptic exon. The *ATM* c.497-2661A > G pathogenic variant (Fig. 2d) resulted in a cryptic exon inclusion through the substitution of A to G in the −1 position of a newly created acceptor site. Similarly, the *BRCA2* c.8332-3384A > T LP variant (Fig. 2e) led to the inclusion of a cryptic exon due to a substitution of A to T in the +2 position of a newly created splice donor site. The *CDH1* intronic deletion (Fig. 2f) likely removed the branch point of the native acceptor, which is predicted to render the native acceptor site unrecognizable to the spliceosome. This deletion also decreased the distance between two preexisting deep intronic cryptic acceptor sites and the 5′ splice donor site. These cryptic acceptor sites were then utilized, causing two different partial intron retention events. Confirmatory analysis via Sanger sequencing or breakpoint analysis was performed for deep intronic variants (Supplemental Fig. 3a–e). These cases, as well as others with deep intronic variants, represent potential false negative results, as the alterations would not have been detected with DNA MGPT sequencing alone.

Here we demonstrate in a large clinical diagnostic cohort of 43,524 individuals that paired DNA- and RNA-seq detects pathogenic variants that impact splicing, including deep-intronic alterations, resulting in the identification of additional 87 individuals with a clinically actionable result. RNA-seq also provided additional evidence for more accurate interpretation of splicing variants, resolving VUS identified in 305 individuals in this cohort alone. Note that results from this study may have underestimated its clinical impact, which can extend well beyond the probands described here, since some of these patients’ families are now eligible for cascade testing. In addition, because reclassified variants may have been detected in previous cases or may be seen in future unrelated individuals, these can have a downstream impact which was also not quantified in this study. In summary, our results highlight the importance of RNA-seq to improve identification of high-risk individuals that would have been missed by DNA-only diagnostic approaches.

## Methods

The authors confirm that we have complied with all ethical regulations and that this study was carried out in accordance with the recommendations of the Western Institutional Review Board (Puyallup, Washington), which has granted an IRB waiver stating “research does not include human subjects” based on federal regulation 45 CFR 46.102(f) and associated guidance entitle, “Guidance on Research Involving Coded Private Information or Biological Specimens”, determining coded private information or biological specimens would not be considered to involve human subjects. All patients described were evaluated by genetic counselors and provided informed consent for testing. Individuals who declined participation in de-identified research were excluded from this study.

Paired DNA and RNA sequencing workflow is depicted in Supplemental Fig. 2. Genomic DNA was isolated from patient’s whole blood or saliva using Qiasymphony (Qiagen). Isolated DNA quantity and quality was assessed using absorbance at 260 nm. A total of 1 μg of genomic DNA was used as input into library prep for dual index sequencing on illumina platforms using a commercially available kit (KAPA Biosystems, Roche). Briefly, DNA was sheared enzymatically, end-repaired, and ligated to standard illumina dual index adapters (IDT). After subsequent library amplification (10 cycles), libraries were pooled together at equal concentrations and target enriched using hybrid capture. Custom-designed biotinylated probes (IDT X-Gen Lockdown) covering the coding regions of tested cancer predisposition genes were hybridized over night to capture libraries and captured with streptavidin beads (LifeTechnologies). Captured libraries were subsequently amplified and prepared for sequencing on NextSeq 500 or NovaSeq using the SP flow cell (illumina). Initial data processing and base calling is performed using the NextSeq Control Software (NCS) and RRTA 2.4.11 (Real Time Analysis NCS v2.0.2.1).

Sequences are aligned to hg19 reference genome and variants are called using the third-party software Genome Analysis Toolkit (GATK) followed by annotation via an internally developed pipeline. While DNA sequencing is routinely performed through a minimum of 30 nucleotides of each intron, masking is applied to variants generated by the bioinformatics pipeline based on the genes included in the test ordered and analytical reporting range (>5 nucleotides beyond each coding exon). Variants with a Q score ≤30 and an allele fraction <10% are filtered out. Regions with <20× coverage on NGS are followed up with Sanger analysis. Variants in regions complicated by pseudogene interference, variant calls not satisfying depth of coverage and variant allele frequency quality thresholds, and potentially homozygous variants are verified by Sanger sequencing. Single nucleotide variants and small insertions/deletions (≤3 nucleotides) that have an allele frequency of >35% and 100× coverage are not verified by Sanger sequencing. Large deletions and duplications are detected using a combination of a read-depth based machine learning method and split-read method, and/or targeted microarray or MLPA as needed.

Total RNA was isolated from an additional patient specimen (blood, PAX tube) using standardized methodology and quantified as described previously^4^. Briefly, total RNA is fragmented and undergoes first strand synthesis using random hexamers, with subsequent ribosomal RNA depletion (Kapa Biosystems, Roche). After second strand synthesis and amplification of libraries ligated with standard illumina dual index adapters. Sequence enrichment of the targeted coding exons and adjacent intronic nucleotides is carried out by a hybrid-capture methodology using long biotinylated oligonucleotide probes followed by a subsequent library amplification and Next-Generation sequencing (NextSeq 500 or Novaseq SP flowcell, illumina).

RNA calls detected on RNA-seq were confirmed via RT-PCRseq if the total coverage for an associated splicing event (PSI denominator) is <500X. In addition, RNA calls with conflicting data, inconsistency in PSI among carriers, or any other quality concerns were also confirmed by RT-PCRseq. For RT-PCRseq, total RNA is converted to complementary DNA (cDNA) by reverse transcriptase polymerase chain reaction (RT-PCR) using a one-step approach with custom-designed primers for the target region (Superscript IV one-step RT-PCR kit, Thermo). Primer sequences are available upon request. RT-PCR amplicons are then library prepped for standard illumina paired end sequencing on MiSeq (illumina) using a commercially available kit (Kapa Hyper Plus Kit, Roche).

RNA samples passed sequencing quality control if the percentage of Q30 bases >75%, mean base quality >30, percentage of perfect index >85%. Reads from samples passing QC were aligned using STAR 2.0 (CITE). An additional QC threshold was applied where ≥85% of exons from the 18 genes have average coverage ≥50×. DNA variants were evaluated for association with abnormal splicing events. Percent Spliced Index (PSI) and its comparison with a control pool of 345 healthy donors were calculated as previously described^1,4^. Relative to the canonical RefSeq transcript isoform annotation, the PSI value was defined as the number of reads supporting the alternative splicing event divided by the number of all reads in the region covering splicing event. Bar plots and box plots were generated using the ggplot2 package (v3.1.1) from R v3.6.1 with default settings. Violin plots were generated using Seaborn (v.0.11.0) within Python v.3.8.3. 5 types of splicing events were considered: Exon Skipping Full (ESF; skipping of at least one full exon), Exon Skipping Partial (ESP; i.e., an alternative 5′ or 3′ splice site that results in exclusion of part of an exon), Exon Skipping Full and Partial (ES; a combination of at least one ESF and ESP), Intron Inclusion Partial (IP; i.e., an alternative 5′ or 3′ splice site that results in inclusion of intronic sequence flanking the exon), and Intron inclusion Cryptic (IC; i.e., a cryptic exon). Technical limitations in our assay may have prevented the detection of full intron retention (IR) events. Association of a splicing event with a DNA variant was evaluated manually by a team of variant assessment scientists. The 18 genes included in RNA sequencing analysis pipeline are: *APC, ATM, BRIP1, BRCA1, BRCA2, CDH1, CHEK2, MLH1, MSH2, MSH6, MUTYH, PALB2, PMS2, PTEN, NF1, RAD51C, RAD51D, TP53*. Reference isoforms are listed in supplemental table 1. Additional considerations: (i) with regards to blood versus tissue samples, the assay has been previously calibrated to identify abnormal splicing in blood by contextualizing putative pathogenic splicing events with previously identified P/LP known to affect splicing^4,5^, additionally the strength of the evidence applied to RNA data for DNA variant curations varies based on blood/tissue expression and alternative splicing identified in controls; (ii) the assay has been previously calibrated to identify NMD-targeted transcripts in blood by contextualizing the PSI of putative pathogenic splicing events versus the PSI of transcripts identified in individuals heterozygous for P/LP variants known to affect splicing and to be targeted by nonsense mediated mRNA decay (NMD)^4,5^. Analyzes of allele skewing using SNPs within exons to indirectly assess NMD is also used depending on SNP availability. Sashimi plots depicted in figures only include reads in the PSI calculation for each abnormal splicing event described.

### Reporting summary

Further information on research design is available in the Nature Research Reporting Summary linked to this article.

## Supplementary information

Supplemental Material

Reporting Summary

## Data Availability

The DNA and RNA sequencing sequence data that support the findings of this study have been deposited in NCBI Sequence Read Archive (SRA) with the submission accession number PRJNA863325. The remaining data that support the findings of this study are available on request from the corresponding author with reasonable patient privacy restrictions.

## Code availability

All analyses conducted are outlined in the Methods section, including software version(s) used. Example code is available upon request.
