# Supplementary material for: Mutational and splicing landscape in a cohort of 43,000 patients tested for hereditary cancer
Source: NPJ Genom Med. 2022 Aug 25;7:49. doi: 10.1038/s41525-022-00323-y (PMC9411123; doi:10.1038/s41525-022-00323-y)
Supplement: Supplementary file 1 — Supplemental Material [file 41525_2022_323_MOESM1_ESM.pdf]

Mutational and splicing landscape in a clinical cohort of 43,000 patients tested for hereditary cancer

Carolyn Horton[1]; Ashley Cass[1]; Blair R. Conner[1]; Lily Hoang[1]; Heather Zimmermann[1]; Nelly Abualkheir[1]; David Burks[1]; Dajun Qian[1]; Bhuvan Molparia[1]; Huy Vuong[1]; Holly LaDuca[1]; Jessica Grzybowski[1]; Kate Durda[1]; Robert Pilarski[1]; Jessica Profato[1]; Katherine Clayback[2]; Martin Mahoney[2]; Courtney Schroeder[3]; Wilfredo Torres-Martinez[3]; Aaron Elliott[4]; Elizabeth Chao[1,5]; Rachid Karam[1]

1) Ambry Genetics. One Enterprise, Aliso Viejo, CA 92656, USA
2) Roswell Park Comprehensive Cancer Center. 665 Elm St, Buffalo, NY 14203, USA
3) Indiana University School of Medicine. 975 W. Walnut Street, IB 130, Indianapolis, IN 46202, USA
4) Realm IDx. One Enterprise, Aliso Viejo, CA 92656, USA
5) University of California, Irvine, School of Medicine. 1001 Health Sciences Rd, Irvine, CA, 92617, USA

Corresponding author:
Rachid Karam, MD PhD
One Enterprise
Aliso Viejo, CA 92656
rkaram@ambrygen.com

Supplemental Figures and Legends

Supplemental Figure 1. Box Plot PSI Distribution by Nucleotide Position in Cases and Controls

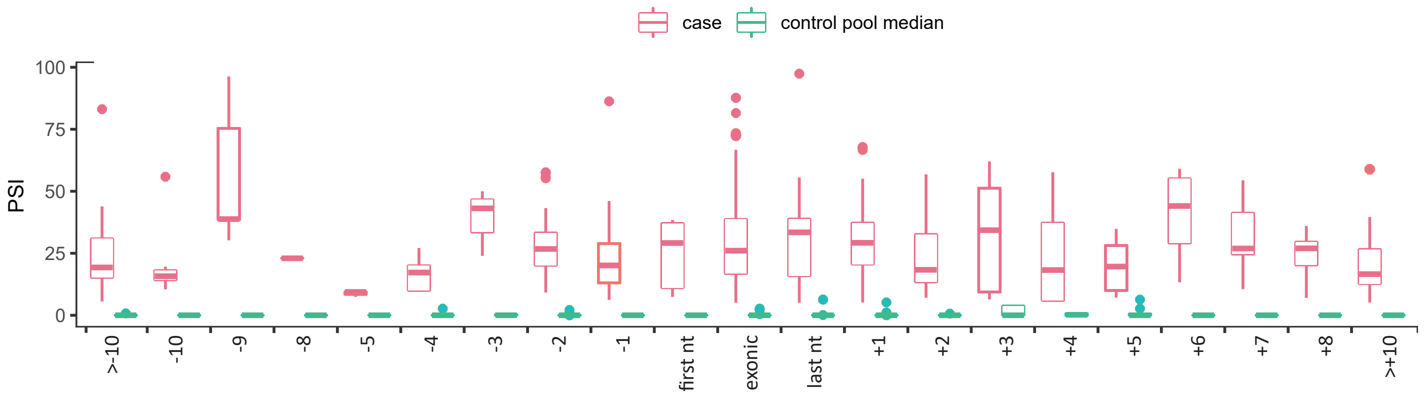

Supplemental Figure 1. Percent spliced index of splicing events associated with variants (P/LP/VUS) in patients (red box plots; N = 516 DNA/RNA associated variants;) and their median in healthy donor controls (teal box plots). Boundaries of each box represent the interquartile range and the error bars represent 1.5x interquartile range.

Supplemental Figure 2. Paired DNA and RNA Sequencing and Interpretation Workflow

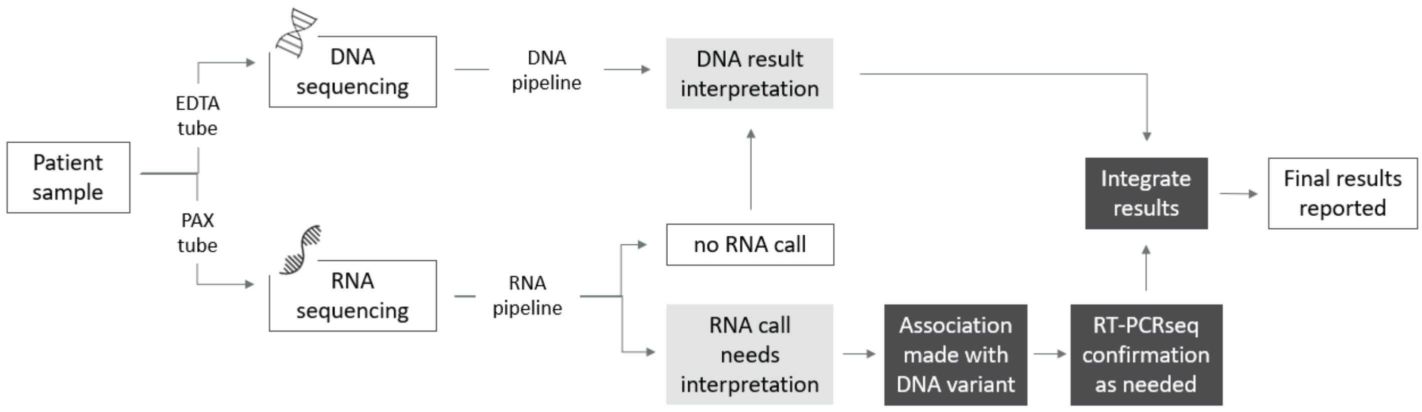

Supplemental Figure 2. Paired DNA and RNA Sequencing and Interpretation Workflow: When RNAinsight is included in a test order, RNAseq is performed on a separate specimen (PAXgene© tube), in parallel with germline DNA sequencing (EDTA tube). Sequencing results are filtered on independent pipelines. Abnormal RNA results undergo manual review to provide association with a corresponding DNA variant. RT-PCRseq is performed as confirmation of RNA result as needed (Supplemental Methods). RNA and DNA results are integrated and any variants are reported with regards to the DNA result

Supplemental Table 1. Reference Sequences for RNA Covered Genes

GENE	Transcript RefSeq
APC	NM_000038.6
ATM	NM_000051.3
BRCA1	NM_007294.3
BRCA2	NM_000059.3
BRIP1	NM_032043.2
CDH1	NM_004360.5
CHEK2	NM_007194.4
MLH1	NM_000249.3
MSH2	NM_000251.2
MSH6	NM_000179.2
MUTYH	NM_001128425.1
NF1	NM_000267.3
PALB2	NM_024675.3
PMS2	NM_000535.7
PTEN	NM_000314.7
RAD51C	NM_058216.9
RAD51D	NM_002878.3
TP53	NM_000546.4

Supplemental Table 1. Reference Sequences for RNA Covered Genes: All sequence analysis is based on the provided NCBI reference sequences

Supplemental Figure 3a. Identification of Deep Intronic Variants

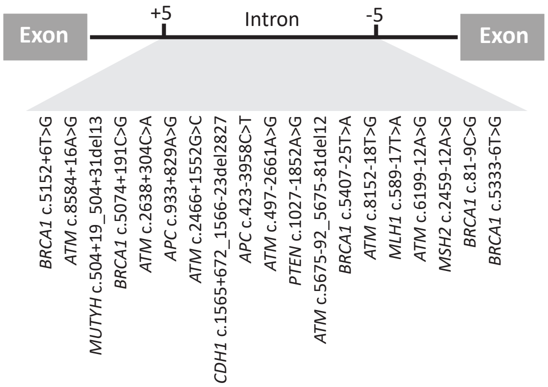

Supplemental Figure 3b. *APC* c.423-3958C>T Sanger Sequencing Results

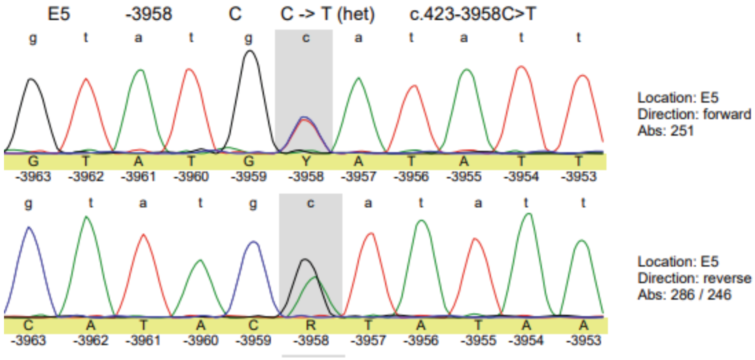

Supplemental Figure 3c. *ATM* c.497-2661A>G Sanger Sequencing Results

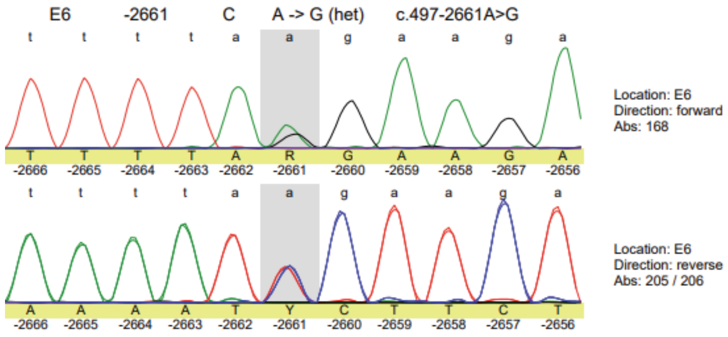

Supplemental Figure 3d. *BRCA2* c.48332-3384A>T Sanger Sequencing Results

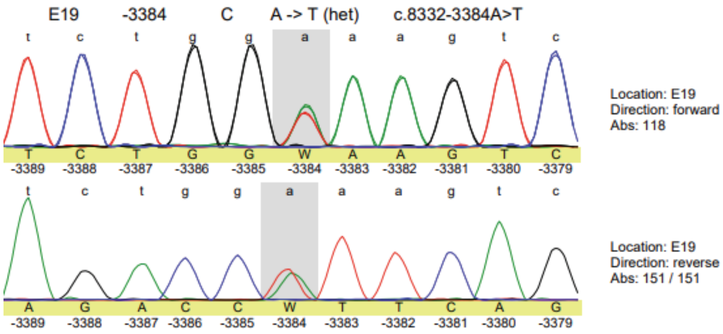

Supplemental Figure 3e. *CDH1* c.1565+672_1566-23del2827 Breakpoint Analysis

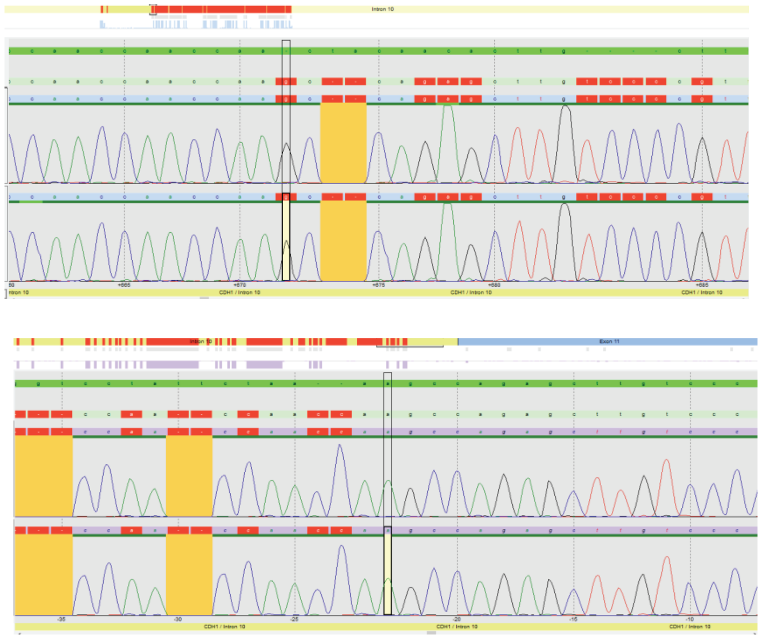

Supplemental Figure 3. Additional Details on Deep Intronic Variants: a) Representation of clinically significant variants identified greater than 5 nucleotides from an exon in the study group. b-e) Confirmatory Analysis of Deep Intronic Variants. b) Bi-directional chromatogram displays the heterozygous C to T substitution at nucleotide position c.423-3958 in *APC*; c) Bi-directional chromatogram displays the heterozygous A to G substitution at nucleotide position c.497-2611 in *ATM*; d) Bi-directional chromatogram displays the heterozygous A to T substitution at nucleotide position c.8332-3384 in *BRCA2*; e) Bi-directional chromatogram displays the results of breakpoint analysis for the intronic gross deletion 1565+672_1566-23del2827 in *CDH1*.
